# Texture analysis of pretreatment [^18^F]FDG PET/CT for the prognostic prediction of locally advanced salivary gland carcinoma treated with interstitial brachytherapy

**DOI:** 10.1186/s13550-019-0555-0

**Published:** 2019-09-11

**Authors:** Wen-Jie Wu, Zhen-Yu Li, Shuang Dong, Shu-Ming Liu, Lei Zheng, Ming-Wei Huang, Jian-Guo Zhang

**Affiliations:** Department of Oral and Maxillofacial Surgery, Peking University School and Hospital of Stomatology, 22# Zhongguancun South Avenue, Beijing, 100081 China

**Keywords:** Texture analysis, PET/CT, Prognosis, Salivary gland carcinoma, Brachytherapy

## Abstract

**Background:**

The aim of this study was to evaluate the prognostic value of positron emission tomography (PET) parameters and the PET texture features of fluorine 18-fluorodeoxyglucose ([^18^F]FDG) uptake on pretreatment PET/computed tomography (CT) in patients with locally advanced salivary gland carcinoma treated with interstitial brachytherapy.

**Methods:**

Forty-three patients with locally advanced salivary gland carcinoma of the head and neck were treated with ^125^I interstitial brachytherapy as the sole modality and underwent [^18^F]FDG PET/CT scanning before treatment. Tumor segmentation and texture analysis were performed using the 3D slicer software. In total, 54 features were extracted and categorized as first-order statistics, morphology and shape, gray-level co-occurrence matrix, and gray-level run length matrix. Up to November 2018, the follow-up time ranged from 6 to 120 months (median 18 months). Cumulative survival was calculated by the Kaplan-Meier method. Factors between groups were compared by the log-rank test. Multivariate Cox regression analysis with a backward conditional method was used to predict progression-free survival (PFS).

**Results:**

The 3- and 5-year locoregional control (LC) rates were 55.4% and 37.0%, respectively. The 3- and 5-year PFS rates were 51.2% and 34.1%, respectively. The 3- and 5-year overall survival (OS) rates were 77.0% and 77.0%, respectively. Univariate analysis revealed that minimum intensity, mean intensity, median intensity, root mean square, and long run emphasis (LRE) were significant predictors of PFS, whereas clinicopathological factors, conventional PET parameters, and PET texture features failed to show significance. Multivariate Cox regression analysis showed that minimum intensity and LRE were significant predictors of PFS.

**Conclusions:**

The texture analysis of pretreatment [^18^F]FDG PET/CT provided more information than conventional PET parameters for predicting patient prognosis of locally advanced salivary gland carcinoma treated with interstitial brachytherapy. The minimum intensity was a risk factor for PFS, and LRE was a favorable factor in prognostic prediction according to the primary results.

## Background

Salivary gland carcinomas are relatively rare malignancies and are diverse, with at least 24 histologic subtypes [1], which brings about challenges in management. Surgery is the mainstay treatment for salivary gland carcinoma, while adjuvant radiotherapy is commonly recommended for cases of advanced stages, gross residual tumors, positive margins, perineural invasion, bone infiltration, or nodal involvement [2–4]. A systematic review revealed that certain drugs may provide palliative effects for a subset of patients with advanced adenoid cystic carcinoma [5]. However, the application and efficacy of chemotherapy on salivary gland carcinoma is limited [2, 4]. For inoperable or locally advanced cases, definitive radiotherapy can be an alternative treatment [3]. As an alternative, interstitial brachytherapy has the advantage of being highly conformal, which results in a high local control in salivary gland carcinoma [6, 7].

Fluorine 18-fluorodeoxyglucose ([^18^F]FDG) positron emission tomography (PET)/computed tomography (CT) is a promising tool for staging and prognosis prediction in many malignancies. Several studies have indicated that pretreatment metabolic [^18^F]FDG PET/CT parameters may predict treatment outcomes in patients with salivary gland carcinomas [8–11]. In addition, texture analysis and other feature extraction algorithms realize the high-throughput extraction of radiomics data from medical images [12]. The texture analysis of PET images was proposed to characterize the heterogeneity of underlying biological processes such as metabolism, hypoxia, cellular proliferation, vascularization, and necrosis [13–15]. The textural features of PET images of primary tumors have been studied for predicting clinical outcome and treatment response in esophageal cancer, colorectal cancer, and cervical cancer [16–18].

In this study, the aim was to evaluate the prognostic value of the conventional PET parameters and PET texture features of [^18^F]FDG uptake on pretreatment PET/CT in patients with locally advanced salivary gland carcinoma treated with interstitial brachytherapy.

## Materials and methods

### Patient characteristics

This retrospective study was approved by the Ethics Committee and was conducted under the guidance of international ethical standards (IRB number: PKUSSIRB-201840168). In this study, 43 patients with locally advanced salivary gland carcinoma of the head and neck were treated with iodine-125 interstitial brachytherapy as the sole modality and underwent [^18^F]FDG PET/CT scan before treatment from March 2008 to May 2018. Patients with a history of radiotherapy or chemotherapy were excluded.

Primary tumors or locoregional recurrent tumors were histologically confirmed by biopsy. Adenoid cystic carcinoma (ACC, 30/43) was the most common histologic subtype of the 43 patients, followed by adenocarcinoma not otherwise specified (NOS) (5/43) and mucoepidermoid carcinoma (3/43). There was one patient for each of the other five types: salivary duct carcinoma, carcinoma in pleomorphic adenoma, myoepithelial carcinoma, basal cell adenocarcinoma, and acinic cell carcinoma. Detailed information regarding characteristics is shown in Table 1. The TNM staging was diagnosed according to the staging criteria of the Union for International Cancer Control (UICC) seventh edition [19]. All the M1 patients were diagnosed with ACC with lung metastasis. Any suspected distant metastatic lesions were histologically confirmed by biopsy or a long-term imaging follow-up.

**Table 1:** Patients characteristics

Characteristics	Number (%)
Sex	
Male	26 (60%)
Female	17 (40%)
Age (years)	
Median	53
Range	25–82
Tumor site	
Major salivary gland	23 (53%)
Minor salivary gland	20 (47%)
T classification	
T4	43 (100%)
N classification	
N0	34 (79%)
N1	2 (5%)
N2	7 (16%)
M classification	
M0	27 (63%)
M1	16 (37%)
Histologic grade	
High grade	5 (12%)
Low grade	38 (88%)
Primary or recurrent tumor	
Primary tumor	22 (51%)
Recurrent tumor	21 (49%)

### Follow-up and evaluation

**Fig. 1:**
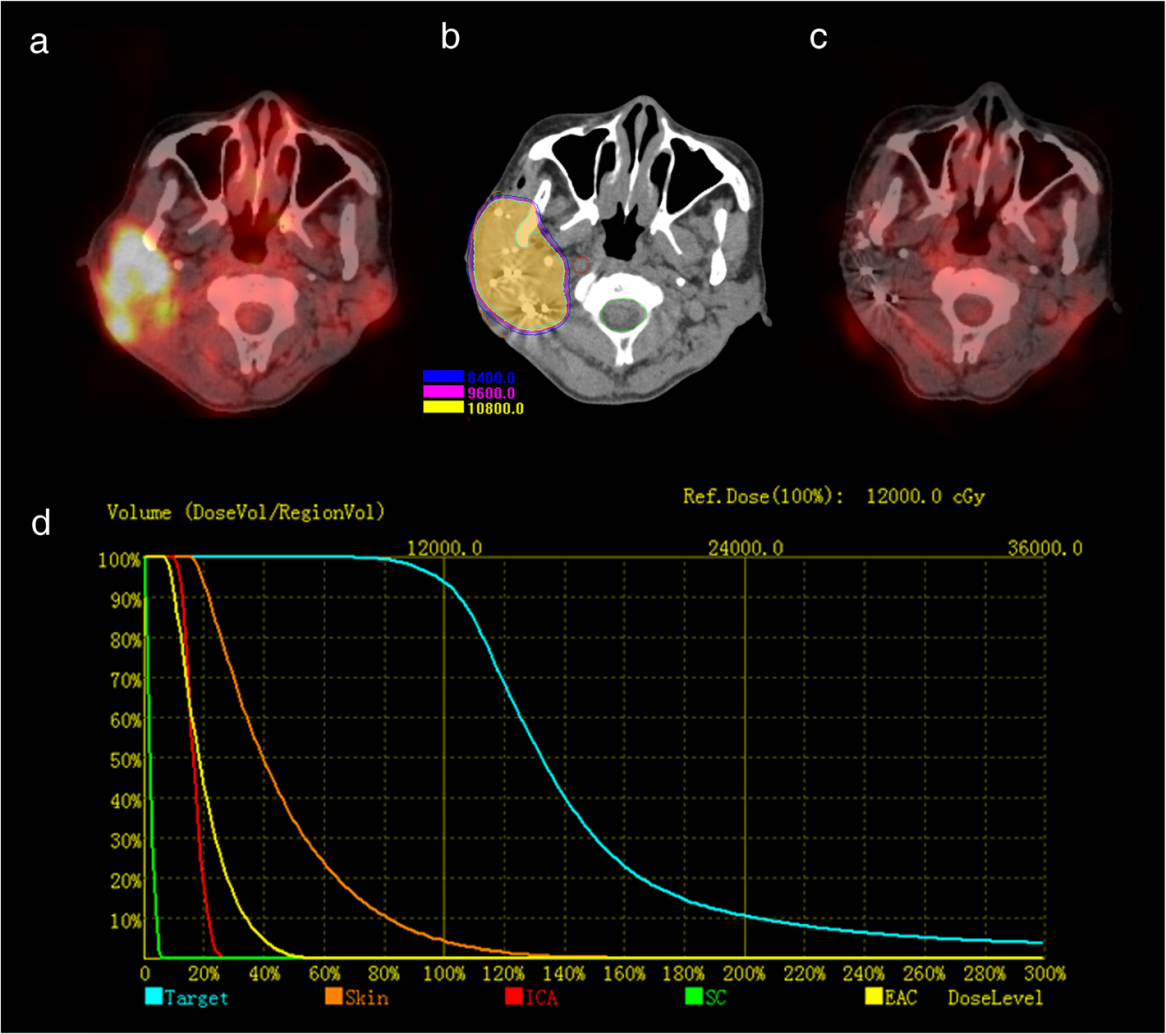
The procedure of brachytherapy based on PET/CT figure: Pretreatment PET/CT showed a T4 parotid gland carcinoma (**a**). Quality verification using postoperative CT images in the brachytherapy treatment planning system showed that the D90 curve covered the tumor area (**b**). PET/CT showed that the area of focal FDG uptake regressed 6 months after brachytherapy (**c**). Dose-volume histograms of the target area and organs at risk are shown (**d**). *ICA* internal carotid artery, *SC* spinal cord, *EAC* external auditory canal

The patients were routinely followed up after brachytherapy at least once every 2 months during the first year. A contrast-enhanced CT (CECT) of the head and neck scan was performed for each follow-up. [^18^F]FDG PET/CT scan was performed when CECT was insufficient for evaluation. The imaging evaluation was based on the Response Evaluation Criteria in Solid Tumors (RECIST) version 1.1 [20]. Treatment response 2–6 months after brachytherapy was classified as complete response (CR) (Fig. 1a, c), partial response (PR), stable disease (SD), or progressive disease (PD). The locoregional control (LC) was defined as the absence of further tumor progression following brachytherapy or the absence of further tumor progression following CR/PR of the primary site. Progression-free survival (PFS) was defined as the period from brachytherapy to the latest follow-up, the absence of LC, or the progression to distant metastasis, withdrawal, or death from any cause. Overall survival (OS) was defined as the period from brachytherapy to the latest follow-up, withdrawal, or death from any cause during the follow-up. The toxicities associated with radiation were recorded and graded according to the Common Terminology Criteria for Adverse Events (CTCAE) version 3.0 [21]. Up to November 2018, the follow-up time ranged from 6 to 120 months (median 18 months).

### Imaging protocols

All patients had a fasting period of a minimum of 6 h and a serum glucose level lower than 160 mg/dL prior to [^18^F]FDG (150–330 MBq) intravenous injection. All patients underwent whole-body PET/CT scan (scanned from vertex to mid-thigh) in the supine position using a Philips Gemini TF16 PET/CT scanner (Philips Medical Systems, Cleveland, OH, USA) with an active axial field of view of 18 cm and an active transverse field of view of 57.6 cm 1 h post injection of [^18^F]FDG. PET images with a 169 × 169 matrix, 4 × 4 × 4 mm^3^ voxel size, and a median of 244 slices (range 215 to 273) were obtained in six bed positions (3 min/bed), along with low-dose CT images with a 512 × 512 matrix, 0.98 × 0.98 × 1.5 mm^3^ voxel size, and a median of 662 slices (range 584 to 740) were obtained for attenuation correction. The total acquisition time was 20 min approximately. Clinical image reconstruction protocols for the Philips Gemini were used, and data were reconstructed using the row-action maximum-likelihood algorithm (RAMLA) 3D with two iterations. In addition, a CECT of the head and neck with a 512 × 512 matrix, 0.4 × 0.4 × 0.75 mm^3^ voxel size, tube voltage of 120 kV, and tube current of 225–300 mA was performed on all patients using a GE Optima CT680.

### ROI segmentation

**Fig. 2:**
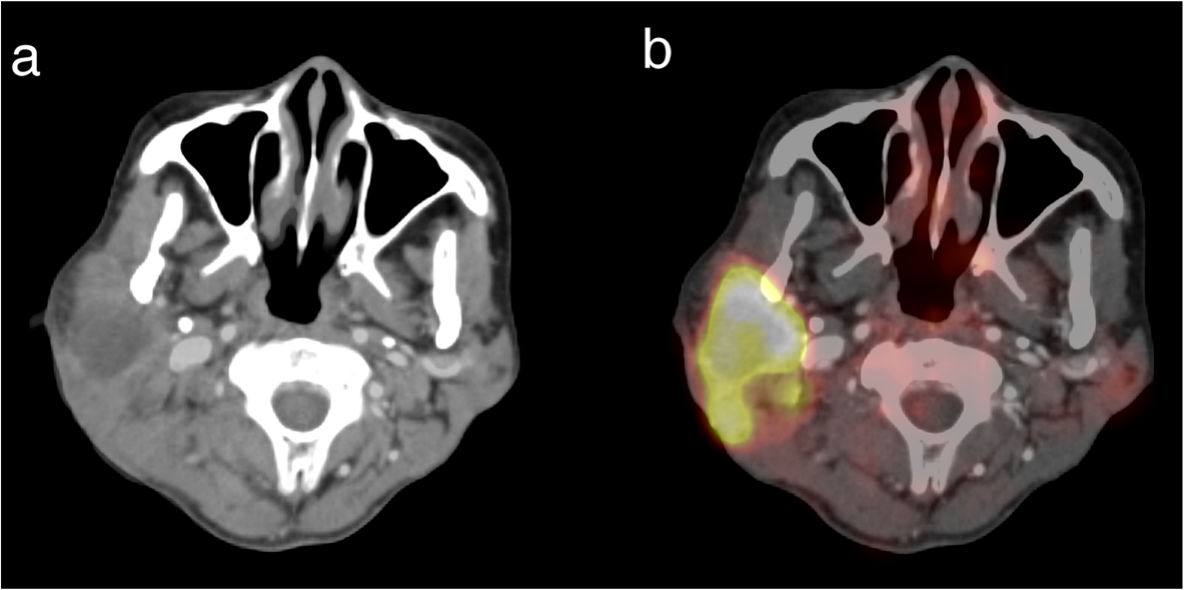
The CT of PET/CT and CECT datasets were registered precisely (**a**). The SUVmax threshold was adjusted based on the registered CECT and the fused images between PET and CECT are shown (**b**)

**Fig. 3:**
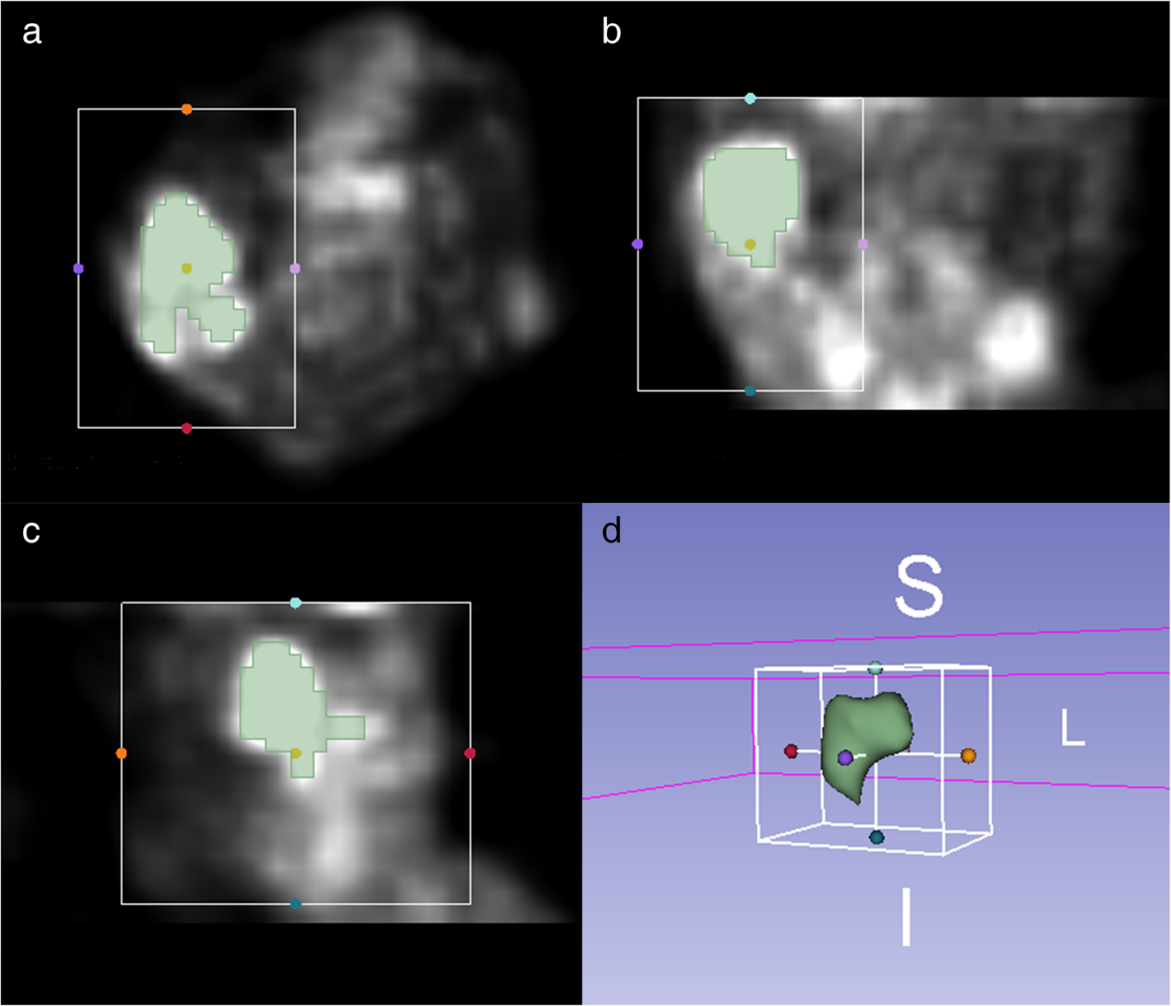
Segmentation of region of interest: Semiautomatic tumor segmentation in 3D slicer was performed on axial (**a**), coronal (**b**), and sagittal (**c**) images. The 3D ROI is shown (**d**)

For all the patients, the matching and registration of images between the CT of PET/CT and CECT datasets were performed automatically on the iPlan CMF 3.0 software (BrainLAB, Feldkirchen, Germany) workstation. Then the maximum standardized uptake value (SUVmax) threshold was adjusted based on the registered CECT (Fig. 2). Semiautomatic tumor segmentation of the registered PET dataset was performed by two radiologists in consensus using a free and open source software (3D slicer, version 4.8.1; available at: http://slicer.org/) [22, 23]. The GrowCut algorithm [24] implemented in 3D Slicer was used to generate the region of interest (ROI) semi-automatically (Fig. 3). After loading the PET images, the process began with user initialization by manually marking the areas inside and outside of the tumor area. First, the high uptake value region and no-uptake value region were roughly delineated manually. Next, the software filled in the high uptake region automatically. Third, the PET images were segmented into foreground ROI and background regions by the GrowCut algorithm. Finally, the background and surrounding isolated foreground pixels were removed, allowing the foreground ROI to be manually edited slightly referring to the PET and CECT, and the texture features to be calculated based on the ROI rather than the background and surrounding parts.

### Texture analysis in 3D slicer

The heterogeneity of each ROI was quantified on a module (https://www.slicer.org/wiki/Documentation/Nightly/Modules/HeterogeneityCAD) [22, 25]. A total of 54 features (Table 2) were extracted and categorized as first-order statistics, morphology and shape, gray-level co-occurrence matrix (GLCM), and gray-level run length matrix (GLRL). The definitions of the features were described in detail in the module. No spatial resampling was performed and the images were discretized using bins of a fixed bin width of 0.5 g/cm^3^. The scale of intensities was set between the minimum and maximum SUV values for each patient, respectively. The neighboring voxel for the co-occurrence matrix in three dimensions were all 1 pixel. Moreover, the metabolic tumor volume (MTV) and the maximum and mean standardized uptake value (SUVmean) of the ROI were calculated and recorded in 3D slicer. SUVmax was defined as the maximum value for SUV in an ROI. MTV was defined as the total lesion volume within a delineated ROI. Total lesion glycolysis (TLG) was defined as the SUVmean multiplied by the MTV, representing an index that includes the metabolic volume and the average uptake within the ROI.

**Table 2:** Classification of texture features

Classification (number)	Features
First-order statistics (14)	Energy, entropy, minimum intensity, maximum intensity, mean intensity, median intensity, range, mean deviation, root mean square, standard deviation, skewness, kurtosis, variance, uniformity
Morphology and shape (8)	Volume (mm^3^), surface area (mm^2^), surface: volume ratio, compactness 1, compactness 2, maximum 3D diameter, spherical disproportion, sphericity
Gray-level co-occurrence matrix (GLCM) (21)	Autocorrelation, cluster prominence, cluster shade, cluster tendency, contrast, correlation, difference entropy, dissimilarity, energy (GLCM), entropy (GLCM), homogeneity 1, homogeneity 2, informational measure of correlation 1 (IMC1), inverse difference moment normalized (IDMN), inverse difference normalized (IDN), inverse variance, maximum probability, sum average, sum entropy, sum variance, variance (GLCM)
Gray-level run length matrix (GLRL) (11)	Short run emphasis (SRE), long run emphasis (LRE), gray level non-uniformity (GLN), run length non-uniformity (RLN), run percentage (RP), low gray level run emphasis (LGLRE), high gray level run emphasis (HGLRE), short run low gray level emphasis (SRLGLE), short run high gray level emphasis (SRHGLE), long run low gray level emphasis (LRLGLE), long run high gray level emphasis (LRHGLE)

### Brachytherapy procedure and parameters

Interstitial brachytherapy was performed with iodine-125 seeds (type 6,711; Atom and High Technique Industries, Beijing, China) that had a half-life of 59.4 days. Radioactive seeds with radioactivity of 18.5–25.9 MBq per seed were used. The preoperative planning and postoperative quality verification were performed in the brachytherapy treatment planning system (BTPS; Atom and High Technique Industries, Inc., Beijing, China). The clinical target volume (CTV) was defined as the gross target volume (GTV) and its surrounding potential subclinical disease that was 0.5 cm beyond the margins [7] of the primary tumor or the positive cervical node based on the CECT which was defined a gold standard to provide accurate anatomical information of head and neck, and the registered PET/CT which provided the additional metabolic information. The planned dose for CTV ranged from 100 to 140 Gy. Brachytherapy was performed under general anesthesia. Iodine-125 seeds were permanently implanted into the target area according to the preplan, with an individual template made via a rapid prototyping technique, and the individual template was constructed with the medial light-cured resin via the rapid forming machine Eden250 (Objet Company, Israel) according to the preplan designed model, combined with CT guidance as demonstrated in detail in a previous study [26, 27].

The amount of the seeds implanted ranged from 17 to 148, with a median of 80, which depended on the target volume and planned dose. As for the actuarial parameters for quality verification using postoperative CT images, the median D_90_ (the dose delivered to 90% of the target volume) of the CTV was 117.57 Gy (102.76–147 Gy), and the median V_100_ (the percentage of the target volume receiving at least 100% of the prescription dose) was 93.6% (90.3–98.6%), whereas the V_150_ (the percentage of the target volume receiving at least 150% of the prescription dose) was < 50% for all patients (Fig. 1b, d).

### Statistical analysis

An optimal cut-off value was calculated using the Youden index from the receiver operating characteristic (ROC) curve analysis of conventional PET parameters (SUVmax, MTV, and TLG). Conventional PET parameters and PET texture features were divided into high-value and low-value groups based on the optimal cut-off values. Then the continuous variables were transformed into binary variables. Cumulative survival was calculated by the Kaplan-Meier method. The clinicopathological factors (tumor site, N classification, M classification, histologic grade, and recurrent tumor), conventional PET parameters, and PET texture features between groups were compared by the log-rank test. Multivariate Cox regression analysis with a backward conditional method was used, including clinicopathological factors, conventional PET parameters, and significant univariate PET texture features to predict PFS. *P* values < 0.05 were considered statistically significant, and all *P* values were two-sided. Bonferroni correction was applied in multivariate Cox regression analysis to reduce the error of type І. The statistical analysis was performed on IBM SPSS Statistics version 20.

## Results

### Tumor response and survival outcomes

During the routine follow-up within 6 months after brachytherapy, 28 patients (65%) developed CR, seven patients (16%) developed PR, five patients (12%) developed SD, and three patients (7%) developed PD. During the subsequent follow-up, four, two, and two patients in the CR, PR, and SD cohort experienced tumor progression, respectively, in addition to the three patients with PD. Among the 11 patients who experienced tumor progression, nine had ACC, one had mucoepidermoid carcinoma, and one had adenocarcinoma NOS. All 11 patients experienced progression at the site of the primary tumor, and two of these patients experienced progression to distant metastasis, while none experienced cervical lymph node metastasis. Three of the 11 patients were lost to follow-up. Five patients died of tumor progression, one died of newly diagnosed lung metastasis, and one died of brain metastasis.

**Fig. 4:**
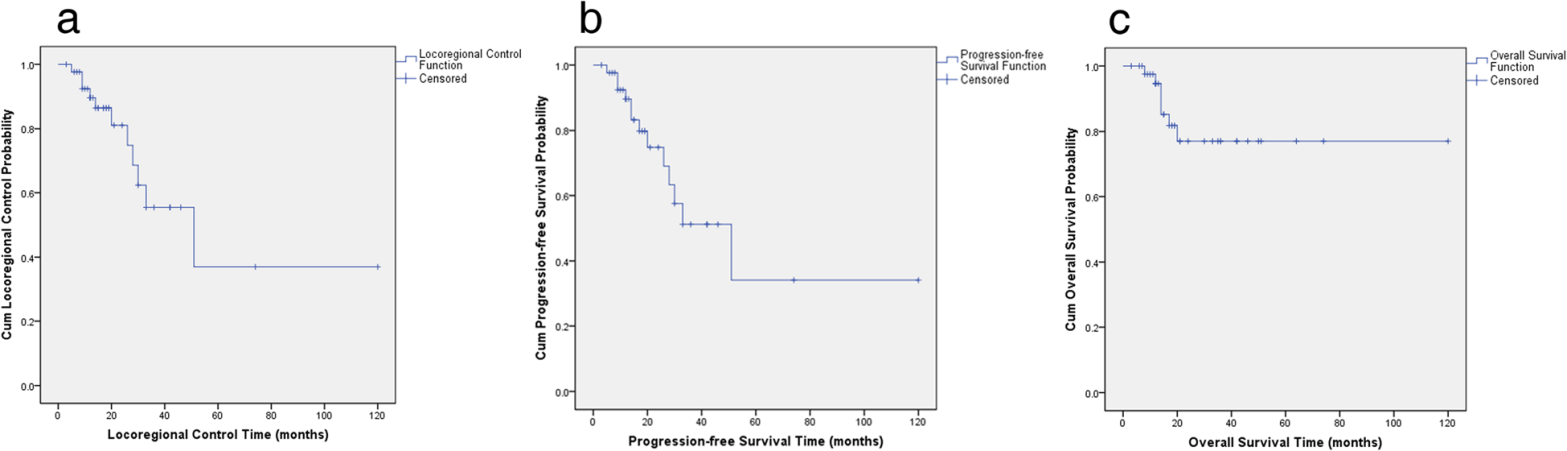
Kaplan-Meier estimates: Kaplan-Meier survival curves of the locoregional control (**a**), progression-free survival (**b**), and overall survival (**c**) are shown

Ten patients with ACC experienced a remission of pain in the tumor region. The 3-year and 5-year LC rates were 55.4% and 37.0%, respectively (Fig. 4a). The 3-year and 5-year PFS rates were 51.2% and 34.1%, respectively (Fig. 4b). The 3-year and 5-year OS rates were 77.0% and 77.0%, respectively (Fig. 4c).

### Toxicities

As for acute toxicities, 15 patients experienced radiodermatitis grade 1–2, and 14 patients experienced mucositis grade 1–2 during the follow-up. Four patients experienced severe pain in the tumor region requiring medical intervention.

As for late toxicities, four patients experienced severe trismus, three patients experienced hemorrhage requiring interventional therapy, and one patient experienced hearing impairment.

### Survival prediction

**Fig. 5:**
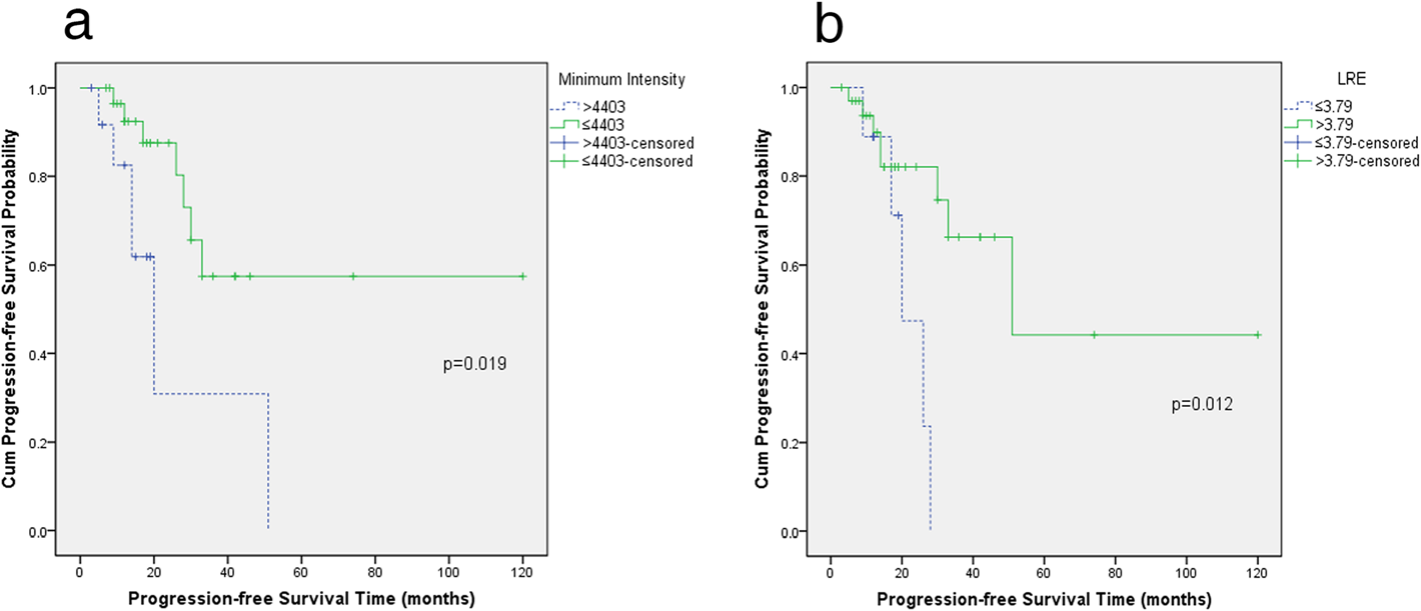
Kaplan-Meier survival curves of the PFS demonstrate significant differences in patients with a high and low minimum intensity (**a**) and long run emphasis (LRE) (**b**)

The optimal cut-off values determined by the ROC curve for the SUVmax, MTV, and TLG were 2.92 g/cm^3^, 57.27 cm^3^, and 37.95 g, respectively. The mean values of the conventional PET parameters and significant texture features showed significant differences between the two groups, respectively (Table 3). And the mean ± 3 × standard deviation did not reach the cut-off values. Univariate analysis revealed that minimum intensity, mean intensity, median intensity, root mean square, and long run emphasis (LRE) were significant predictors of PFS (*P* < 0.05), whereas clinicopathological factors, conventional PET parameters, and PET texture features failed to show significance (Table 3 and Fig. 5).

**Table 3:** Log-rank tests of clinicopathologic factors, conventional PET parameters, and significant texture features for PFS

Factors or parameters	Number (%)	Value of parameters (mean ± standard deviation)	3-year PFS rates	*P* value
Tumor site				0.057
Major salivary gland			72.3%	
Minor salivary gland			31.5%	
N classification				0.122
N0			56.1%	
N+			44.4%	
M classification				0.217
M0			61.9%	
M1			32.6%	
Histologic grade				0.855
High grade			33.2%	
Low grade			80.0%	
Primary or recurrent tumor				0.352
Primary tumor			63.6%	
Recurrent tumor			41.5%	
SUVmax (g/cm^3^)				0.455
≤ 2.92	15 (35%)	2.36 ± 0.10	37.1%	
> 2.92	28 (65%)	6.27 ± 0.54	67.6%	
MTV (cm^3^)				0.496
≤ 57.27	33 (77%)	22.46 ± 2.45	59.6%	
> 57.27	10 (23%)	101.52 ± 9.09	34.3%	
TLG (g)				0.246
≤ 37.95	14 (33%)	21.39 ± 2.54	50.2%	
> 37.95	29 (67%)	133.61 ± 18.09	56.7%	
Minimum intensity				0.019*
≤ 4403	30 (70%)	2813.37 ± 178.48	57.5%	
> 4403	13 (30%)	6017.31 ± 414.55	0	
Mean intensity				0.027*
≤ 6328	25 (58%)	4015.43 ± 258.37	61.1%	
> 6328	18 (42%)	9426.24 ± 639.15	23.7%	
Median intensity				0.027*
≤ 6119	26 (60%)	3815.35 ± 254.19	61.1%	
> 6119	17 (40%)	8942.24 ± 598.51	23.7%	
Root mean square				0.027*
≤ 6431	26 (60%)	4119.35 ± 262.72	61.1%	
> 6431	17 (40%)	9752.68 ± 681.31	23.7%	
Long run emphasis				0.012*
≤ 3.79	34 (79%)	2.17 ± 0.13	0	
> 3.79	9 (21%)	5.96 ± 0.93	66.3%	

*PET* positron emission tomography, *PFS* progression-free survival, *SUVmax* maximum standardized uptake value, *MTV* metabolic tumor volume, *TLG* total lesion glycolysis, "*" represents *P*<0.05

Multivariate Cox regression analysis showed that minimum intensity and LRE were significant predictors of PFS (*P* < 0.05) (Table 4). The PFS rate was relatively higher in patients with a lower minimum intensity and a higher LRE.

**Table 4:** Multivariate Cox regression analysis of significant texture features for PFS

Factors or parameters	Hazard ratio (95% confidence interval)	*P* value
Minimum intensity	3.577 (1.129–11.327)	0.030
Long run emphasis	0.231 (0.065–0.825)	0.024

*PFS* progression-free survival\

The ROC curve showed that the area under the curve (AUC) for minimum intensity was 0.614 (95% CI 0.424–0.804), and the sensitivity and specificity were 76.7% and 53.8%, respectively. The AUC for LRE was 0.626 (95% CI 0.433–0.819), and the sensitivity and specificity were 86.7% and 61.5%, respectively.

## Discussion

The management of locally advanced salivary gland carcinoma with known radio-resistance remains challenging and controversial. As one of the major histologic subtypes of salivary gland carcinomas, ACC is an aggressive histologic subtype with high metastatic potential. Therefore, several reports have shown concern about the management of ACC (Table 5). Definitive radiotherapy can be considered as a modality in inoperable cases or macroscopic residual disease after surgery and particle radiotherapy for locally advanced salivary gland carcinoma has also achieved favorable results [3]. The risk factor for long-term survival was distant metastasis due to the high rates of distant metastasis of salivary gland carcinoma [3, 28–31]. Nevertheless, in our study, locally advanced salivary gland carcinoma may still be considered a curative entity in the presence of the long-term stable lung metastasis of certain subtypes of salivary gland carcinoma, which was consistent to some literature [32, 33]. Moreover, only one patient with ACC died of newly diagnosed lung metastasis in this cohort during the follow-up, while the other 16 patients survived with lung metastatic lesions. And among the 11 patients who experienced tumor progression, nine had ACC. Tumor progression due to perineural spread is one of the biological characteristics of ACC [34]. The advantages of interstitial brachytherapy include being completed at one time under general anesthesia and being highly conformal, especially for tumors located in deep regions of the head and neck. A total of 38 patients with locally advanced ACC were treated with interstitial brachytherapy according to Huang et al. [35], and the results showed that the 5-year local control and OS rates were 59% and 65%, respectively. For inoperable parotid gland carcinomas, the 5-year local control and OS rates were 58.2% and 61%, respectively in the literature [7], and the results revealed that the treatment efficacy of interstitial brachytherapy for locally advanced salivary gland carcinoma was comparable to that of particle radiotherapy. In the present study, the treatment efficacy showed a promising response to interstitial brachytherapy in locally advanced salivary gland carcinoma. The relatively low long-term LC and PFS rates in our study were likely due to patients with advanced TNM stages. Overall, encouraging treatment efficacy was achieved compared to the findings reported in the literature (Table 5) [29–31, 36–40]. The dose of radiation exposure to the patient was difficult to estimate; however, the dose of radiation exposure was mainly from brachytherapy and PET/CT scan in our study. The mean effective dose equivalent of waist and collar were 6.47 mSv and 1.10 mSv, respectively in a group of 29 patients treated with prostate brachytherapy with a prescription of 145 Gy and a mean activity of 1402.3 MBq iodine-125 [41]. The total effective dose to the patient from a PET/CT procedure can reach 10 mSv [42]. However, radiation therapy and PET/CT scan were commonly used in the area of head and neck oncology especially for advanced-stage malignancies despite the radiation exposure.

**Table 5:** Radiotherapy for locally advanced salivary gland carcinomas

First Author, Year	Cancer type	No. of patients	Treatment	LC rates (5-, 10-year)	PFS rates 5-year	OS rates 5-, 10-year
Mendenhall, 2004 [36]	T4 ACC	42	Radiotherapy	44%, 30%	/	50%, 29%
Chen, 2006 [37]	T4 salivary gland carcinomas	12	Radiotherapy	42%, 30%	/	42%, 37%
Douglas, 2000 [38]	Locally advanced ACC	151	fast Neutron radiotherapy	57%, /	/	72%, /
Gentile, 2017 [29]	Nasopharynx ACC involving the skull base	14	Proton beam therapy	/	/	59%, /
Saitoh, 2017 [39]	Inoperable adenocarcinomas	47	Carbon-ion radiotherapy	79.3%, /	/	43.4%, /
Jensen, 2015 [40]	Inoperable or subtotally resected ACC	58	Intensity-modulated radiotherapy (IMRT) and carbon-ion therapy	59.6%, /	48.4%, /	76.5%, /
Jensen, 2015 [30]		37	Photon therapy	39.9%, /	27%, /	58.7%, /
	Inoperable malignant salivary gland tumors	16	Carbon-ion therapy followed by IMRT	(3-year) 75, /	(3-year) 49.2%, /	(3-year) 74.5%, /
Takagi, 2014 [31]	Inoperable ACC	56	Proton therapy or carbon-ion therapy alone	66%, /	34%, /	51%, /

*ACC* adenoid cystic carcinoma, *LC* local control, *PFS* progression-free survival, *OS* overall survival

Several studies have indicated that [^18^F]FDG PET/CT is an important tool for staging, restaging, and treatment outcome prediction in patients with salivary gland carcinomas [9–11]. PET conventional parameters such as SUVmax, MTV, and TLG were important parameters in the outcome prediction of tumors despite the limitation of heavily depending on the PET scanner and reconstruction methods. Hsieh et al. [9] reported that 46 patients with salivary gland carcinoma were treated with definitive IMRT, and the 5-year PFS and OS rates were 63% and 61%, respectively. The results also indicated that pretreatment SUVmax on [^18^F]FDG PET/CT images predicted outcomes in this group of patients. Moreover, ROI segmentation or MTV definition based on PET images remains a challenge especially for salivary gland carcinoma. A fixed threshold of SUVmax for tumor delineation was set in several studies [10, 43]. However, there was a direct relationship between ROI and threshold of SUVmax. In our study, as for salivary gland carcinoma with a lower SUVmax such as ACC, the ROI segmentation was segmented semi-automatically based on the registered PET. Moreover, ROI segmentation was performed by two radiologists in consensus based on registered PET, and the precision of texture feature measurements was not evaluated in our study, despite that smoothing and segmentation have only a small effect on the precision according to the PET data of oesophageal cancer [44]. In the literature, a study showed that MTV and TLG were independent prognostic factors in patients with intermediate or high-grade salivary gland carcinomas [10]. However, Park et al. [45] concluded that the diagnostic accuracy of PET/CT for evaluating tumor extent was comparable to that of conventional imaging studies and offered no additional advantage for detecting locoregional recurrence. As a noninvasive method that can provide a wealth of characteristic information, texture analysis has been applied in several tumor areas, including head and neck cancer [15]. However, very few studies have focused on the prognostic value of texture analysis in patients with salivary gland carcinoma.

Many challenges, including consensus, reproducibility, and standardization, remain in the field of texture analysis. Several studies have focused on the prognostic value of the heterogeneity parameters in patients with head and neck cancer like oropharyngeal cancer [46–48] or hypopharyngeal cancer [49]. As for salivary gland carcinoma, Cheng et al. investigated the prognostic value of PET/CT texture features and parameters in salivary gland carcinoma and concluded that SUVmax and SUV entropy were associated with the prognosis of salivary gland carcinoma [43]. In the present study, minimum intensity, one of the first-order statistics features, and LRE, one of the GLRL features, were significant factors of PFS in patients with locally advanced salivary gland carcinoma treated with interstitial brachytherapy. Minimum intensity represents the lowest value of the voxels in the ROI, and LRE represents the highest value indicative of longer run lengths and coarser structural texture, which may characterize the underlying tumor heterogeneity. The different results of texture analysis between our study and the study by Cheng et al. may be mainly due to PET reconstruction methods, ROI segmentation, and the relatively small sample size. Moreover, the SUVmax was not significantly associated with prognosis in our study, which is inconsistent with that reported in the literature [10, 43]. This conflicting result may be because patients with ACC account for the vast majority of all the patients in this study and because ACC is characterized by low FDG avidity. In other words, for tumors with a lower SUVmax such as ACC, we require a more reliable method to predict prognosis. Texture analysis is an alternative to conventional analysis based on conventional PET parameters. The relatively small sample size and lack of validation cohort were limitations of this study due to the low incidence of salivary gland carcinoma. Multivariate Cox regression analysis was performed using the transformed binary variables instead of the continuous variables in this cohort with a relatively small sample size, and Bonferroni correction was applied to reduce the error of type І. Moreover, the correlation of texture features and histologic manifestations remains unknown and to be clarified, and prudent attitude should be made toward the significant and insignificant features. Nevertheless, texture analysis may play a more important role in prognostic prediction and clinical strategy in the future.

## Conclusions

The texture analysis of pretreatment [^18^F]FDG PET/CT provided more information than conventional PET parameters in predicting the prognosis of locally advanced salivary gland carcinoma treated with interstitial brachytherapy. The minimum intensity was a risk factor for PFS, and LRE was a favorable factor in prognostic prediction according to the primary results.

## Data Availability

The datasets used and/or analyzed during the current study are available from the corresponding author on reasonable request.

## Abbreviations

[^18^F]FDG: Fluorine 18-fluorodeoxyglucose
ACC: Adenoid cystic carcinoma
AUC: Area under the curve
CECT: Contrast-enhanced CT
CR: Complete response
CTCAE: Common Terminology Criteria for Adverse Events
CTV: Clinical target volume
GLCM: Gray-level co-occurrence matrix
GLRL: Gray-level run length matrix
GTV: Gross target volume
Gy: Gray
kV: Kilovolt
LC: Locoregional control
LRE: Long run emphasis
mA: Milliampere
MBq: Megabecquerel
mSv: Millisievert
MTV: Metabolic tumor volume
NOS: Not otherwise specified
OS: Overall survival
PD: Progressive disease
PET/CT: Positron emission tomography/computed tomography
PFS: Progression-free survival
PR: Partial response
RECIST: Response Evaluation Criteria in Solid Tumors
ROC: Receiver operating characteristic
ROI: Region of interest
SD: Stable disease
SUVmax: Maximum standardized uptake value
SUVmean: Mean standardized uptake value
TLG: Total lesion glycolysis
UICC: Union for International Cancer Control
